# Immune-related SERPINA3 as a biomarker involved in diabetic nephropathy renal tubular injury

**DOI:** 10.3389/fimmu.2022.979995

**Published:** 2022-10-11

**Authors:** Zuyan Fan, Yan Gao, Nan Jiang, Fengxia Zhang, Shuangxin Liu, Quhuan Li

**Affiliations:** ^1^ School of Bioscience and Bioengineering, South China University of Technology, Guangzhou, China; ^2^ Guangdong Provincial Engineering and Technology Research Center of Biopharmaceuticals, South China University of Technology, Guangzhou, China; ^3^ Department of Nephrology, School of Medicine, South China University of Technology, Guangzhou, China; ^4^ Department of Nephrology, Guangdong Provincial People’s Hospital, Guangdong Academy of Medical Sciences, Guangzhou, China; ^5^ Department of Nephrology, First Affiliated Hospital of Gannan Medical University, Ganzhou, China; ^6^ The Second School of Clinical Medicine, Southern Medical University, Guangzhou, China

**Keywords:** diabetic nephropathy, renal tubules, RNA-Seq, bioinformatic, SERPINA3

## Abstract

Diabetic nephropathy (DN) is the leading cause of end-stage renal disease and has become a serious medical issue globally. Although it is known to be associated with glomerular injury, tubular injury has been found to participate in DN in recent years. However, mechanisms of diabetic renal tubular injury remain unclear. Here, we investigated the differentially expressed genes in the renal tubules of patients with DN by analyzing three RNA-seq datasets downloaded from the Gene Expression Omnibus database. Gene set enrichment analysis and weighted gene co-expression network analysis showed that DN is highly correlated with the immune system. The immune-related gene SERPINA3 was screened out with lasso regression and Kaplan–Meier survival analyses. Considering that SERPINA3 is an inhibitor of mast cell chymase, we examined the expression level of SERPINA3 and chymase in human renal tubular biopsies and found that SERPINA3 was upregulated in DN tubules, which is consistent with the results of the differential expression analysis. Besides, the infiltration and degranulation rates of mast cells are augmented in DN. By summarizing the biological function of SERPINA3, chymase, and mast cells in DN based on our results and those of previous studies, we speculated that SERPINA3 is a protective immune-related molecule that prevents renal tubular injury by inhibiting the proliferation and activation of mast cells and downregulating the activity of chymase.

## Introduction

Diabetic nephropathy (DN) is a serious global medical problem. Damage tends to be located in the glomerulus, renal tubules, and renal interstitium. Approximately 25–40% of patients with type 1 diabetes and 5–40% of patients with type 2 diabetes eventually develop DN (1, 2). DN is the leading cause of end-stage renal disease, causing 30–50% of cases (3). Although DN is primarily considered to result in glomerular injury, currently, an increasing number of studies on diabetic renal injury are revealing that tubular injury plays a critical role in the development of DN (4–7). However, the mechanisms of tubular injury in DN have not yet been elucidated. In addition, the levels of markers of renal tubular injury detected in urinary tubules, such as those of neutrophil gelatinase-associated lipoprotein, kidney injury molecule 1, liver-type fatty acid binding protein, N-acetyl-β-glucosidase, and retinol-binding protein, may be increased in patients with diabetes prior to the appearance of microalbuminuria. This suggests that renal tubular injury plays an important role in DN (6, 7). Therefore, it is of great significance to clarify the pathogenesis of DN to design appropriate treatment strategies.

With the development of nephropathy research, many DN pathogenic mechanisms have been proposed. One hypothesis is that renal cells can maintain cell survival and renal integrity through basal autophagy, whereas in diabetic renal cells, disordered renal autophagy and increased oxidative stress levels are caused by the deregulated Akt/mTOR signaling pathway, leading to renal injury (8). In addition, some researchers believe that extracellular vesicles, especially exosomes and microvesicles, are involved in the pathological process of DN. Under high glucose stimulation, kidney cells abnormally secrete extracellular vesicles. These extracellular vesicles, which are rich in various proteins, lipids, and mRNAs, mediate communication between cells. For example, TGF-β1 mRNA is transported through extracellular vesicles. It activates TGF-β1/Smad3, Wnt/β-catenin, TGF-β1/PI3-Akt, and other signaling pathways in renal cells, which induce epithelial interstitial transformation, podocyte apoptosis, proximal tubular cell injury, and other renal lesions, ultimately inducing DN (9). In addition, accumulating evidence indicates that a large number of proinflammatory molecules and pathways are involved in the occurrence and development of DN, such as the transcription factor NF-κB, CCL2, CX3CL1, other chemokines, IL-1, IL-6, tumor necrosis factor, and other inflammatory cytokines. The discovery of these mediators of pathogenesis will contribute to the development of new treatments for DN from the perspective of anti-inflammation (10).

**Figure 1:**
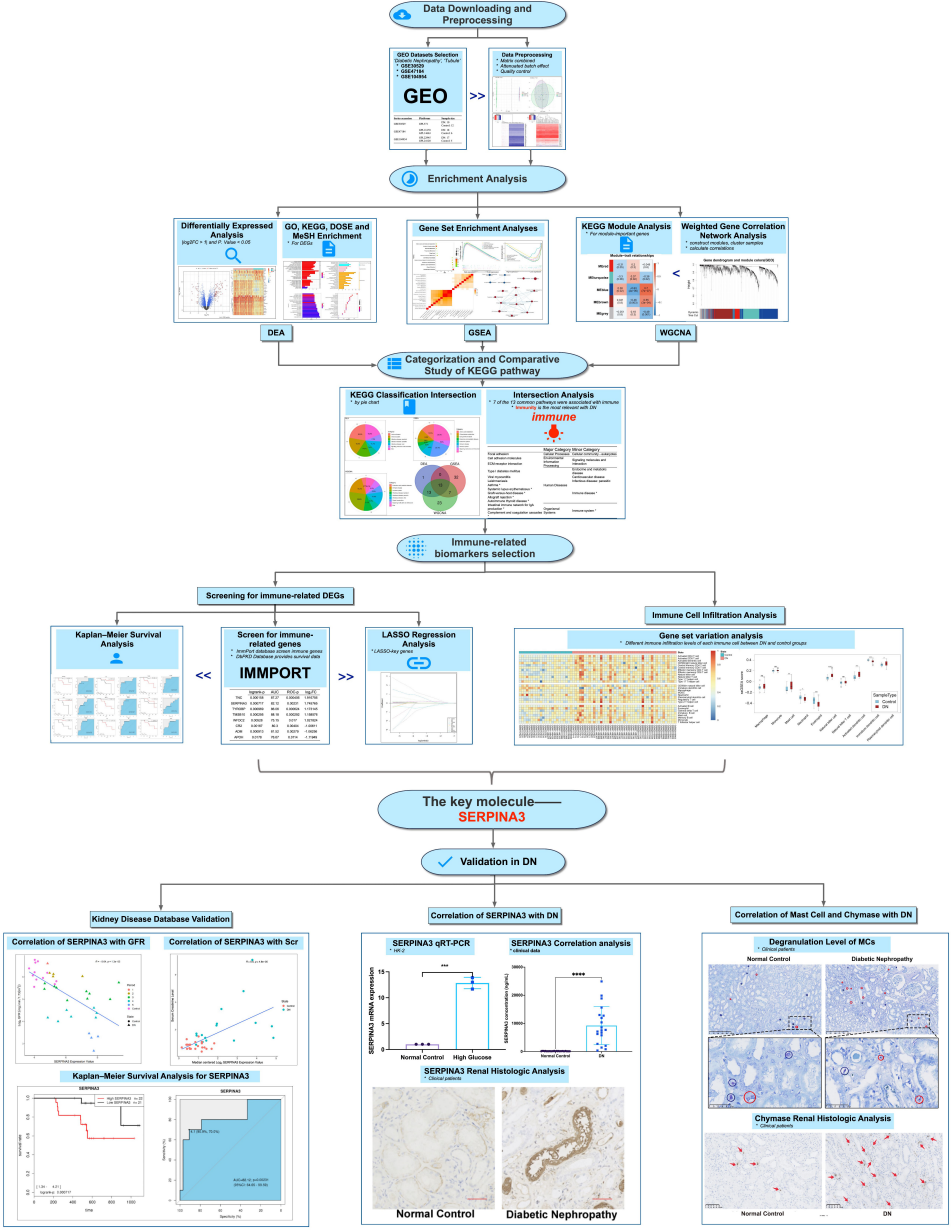
Workflow of the study. *P < 0.05, **P < 0.01, ***P < 0.001, and ****P < 0.0001; ns, no significance.

To elucidate the pathogenesis of DN and search for new biomarkers of diabetic renal tubular injury, a comprehensive bioinformatics analysis of transcriptional sequencing data from renal tubule DN samples was conducted (**Figure 1**). First, 13 genes with the highest correlation with DN were screened by differential expression, weighted gene co-expression network, and gene set enrichment analyses. Lasso regression and Kaplan–Meier survival analyses were used to identify potentially novel molecules. Combined with immune infiltration analysis, SERPINA3 was identified as a potentially valuable biomarker. Subsequently, the relationship between the expression levels of SERPINA3 in renal tubules and glomerular filtration rate (GFR) and serum creatinine level was studied based on the clinical information of patients with DN obtained from the Nephroseq database. Finally, based on the correlation between SERPINA3 expression and the activity of chymase secreted from mast cells, a theory relating to the pathogenesis of diabetic renal tubular injury was developed. This study identified the immune-related molecule SERPINA3 as a possible new marker of diabetic renal tubular injury.

## Materials and methods

### Data collection and differential gene expression analyses

Microarray series from the Gene Expression Omnibus (GEO) database were searched using the following keywords: diabetic nephropathy, tubule, and *Homo sapiens*. Three datasets (GSE30529, GSE47184, and GSE104954) were downloaded to select the required samples. Here, 12 DN and 10 normal control tubule samples from GSE30529, 18 DN and 6 tumor nephrectomy samples from GSE47184, and 17 DN and 5 tumor nephrectomy samples from GSE104954 were selected. Tumor nephrectomy samples were taken as normal controls. Summarized information of the analyzed data is provided in **Table 1**. Probe IDs were converted into the corresponding official gene names to annotate the expression matrices. For genes matching several probes, the probe with the maximum expression value was chosen. The three expression matrices were then merged into one. The batch effects were removed *via* the R package *sva* to obtain a merged expression matrix including 45 DN samples and 23 control samples. Principal component analysis, clustering, and heatmaps were used to visualize the merged matrix before and after batch effect removal.

**Table 1:** Summary of diabetic nephropathy studies on kidney tubules and associated microarray datasets from the Gene Expression Omnibus database.

Series accession	Platform	Sample size
GSE30529	GPL571	DN: 10, NC: 12
GSE47184	GPL11670, GPL14663	DN: 18, NC: 6
GSE104954	GPL22945, GPL24120	DN: 17, NC: 5

DN, diabetic nephropathy; NC, normal control.

The Package *limma* (11) was applied to distinguish differentially expressed genes (DEGs) between DN and control samples with the criteria of |log_2_FC| > 1 and P value < 0.05. The packages *pheatmap* and *EnhancedVolcano* were used to visualize the DEGs.

### Enrichment analyses for DEGs

The packages *clusterProfiler* (12) and *org.Hs.eg.db* (13) were applied to perform Gene Ontology (GO) and Kyoto Encyclopedia of Genes and Genomes (KEGG) pathway enrichment analyses, and the packages *DOSE* (14) and *meshes* were used to perform Disease Ontology Semantic and Enrichment (DOSE) analysis and Medical Subject Heading (MeSH) enrichment analysis of DEGs. Results with a Q value of ≤ 0.05 were considered significant. The results were visualized in the form of a bar chart and dot plot using the packages *ggplot2* (15) and *enrichplot*. The results of KEGG pathway enrichment analyses were categorized according to information from the KEGG pathway database (https://www.kegg.jp/kegg/pathway.html).

### Gene set enrichment analysis (GSEA) and weighted gene correlation network analysis (WGCNA)

Using GSEA software (version 4.1.0), enrichment analyses were conducted on the merged expression matrix using *c2.cp.kegg.v7.4. symbols.gmt* was used as a reference gene set. Pathways with a false discovery rate (FDR) of < 0.25 and a normalized P value of < 0.05 after 1,000 permutations were considered statistically significant. The results were visualized using the R package *ggplot2*. Leading edge analysis and enrichment map visualization were conducted for enrichment analysis results obtained using GSEA software.

The R package *WGCNA* (16) was used to construct the modules. First, the genes with the top 25% expression variance were selected for WGCNA. Second, the samples were clustered to detect outliers. Third, a soft thresholding power of 10 was selected to construct the adjacency matrix, which was then transformed into a topological overlap matrix (TOM). Genes were categorized into multiple gene modules according to TOM-based dissimilarity. Modules were clustered to detect similar modules, which were merged into one using the R function *mergeCloseModules*. Correlations between the modules and clinical traits were calculated and visualized. Gene significance for DN, module membership (MM) in most DN-related modules, and the Pearson correlations between them were calculated. MM values in the upper quartile of all genes in the blue module were selected as module-important genes, and their functional annotation was investigated through KEGG pathway enrichment analyses.

Based on category information from the KEGG pathway database, the results of KEGG pathway enrichment analyses for DEGs, GSEA, and module-important genes were categorized and visualized in pie charts. Next, the intersection of the three KEGG pathway enrichment results was used to observe the common enrichment pathways and their characteristics.

### Immune-related DEG (IRDEG) collection and hub gene selection

A list of 2,483 immune-related genes (IRGs) was downloaded from the ImmPort database. A total of 32 IRDEGs were identified by the intersection of IRGs and DEGs. dbPKD, a database for prognostic markers of kidney diseases, can assess the effect of user-provided genes on survival. IRDEGs were submitted to dbPKD to perform Kaplan–Meier survival analysis to assess their potential as prognostic markers. The logrank P value was < 0.05 and ROC-P statistical significance was set at P < 0.05. These genes were identified as potential prognostic genes. Key genes were selected from the IRDEGs by lasso regression analysis using the R package *glmnet* (17). Then, the intersection of lasso key genes with potential prognostic genes was obtained to identify hub genes.

To verify the relationship between hub gene expression and the clinical characteristics of DN, clinically relevant data were obtained from Nephroseq v5 (https://v5.nephroseq.org), and hub gene expression between patients with DN and healthy controls was analyzed. The differences between the two groups were calculated using the Wilcoxon test. Statistical significance was defined as a two-tailed P value of < 0.05.

### Evaluation of immune cell infiltration

Single-sample GSEA (ssGSEA) was used to assess the extent of immune infiltration in the samples. The R package *GSVA* was used for analysis. TISIDB is an integrated repository portal for tumor–immune system interactions, and 28 characteristic gene sets of infiltrating immune cells were downloaded from here for ssGSEA. After standardization of the gene expression matrix, *GSVA()* was used for analysis. The parameters were set as follows: method = ‘ssGSEA, KCDF = ‘Gaussian, abs., and Ranking = TRUE. Then, Student’s *t*-test was conducted on the ssGSEA score of immune cells between the DN and control groups. Finally, the ssGSEA score was normalized to optimize the visualization.

### Cells and patient tissues

The immortalized human kidney proximal tubular epithelial cell line HK-2 was purchased from the American Type Culture Collection (ATCC, Manassas, VA, USA). The cells were incubated in a constant-temperature incubator at 37°C and 5% CO_2_. HK-2 cells were divided into two groups: normal glucose (NG) and high glucose (HG). All cells in the HG group were cultured in DMEM containing 30.0 mmol/L glucose and incubated for the corresponding time, whereas cells in the NG group were cultured in DMEM with 5.3 mmol/L glucose.

Samples from four patients with DN and four healthy individuals were donated by the Department of Nephrology, Guangdong Provincial People’s Hospital. Each sample included peritubular kidney tissue. Pathological and clinical follow-up data were collected for all cases after ethical approval from the ethical review committee. In addition, serum from 12 patients with DN and 83 healthy individuals as well as urine samples from 20 patients with DN and 44 healthy individuals were collected to measure the proteinuria and creatinine levels.

### SERPINA3 expression levels in cells and blood and urine samples

TRIzol reagent (Invitrogen) was used to extract total RNA from cells, followed by reverse transcription of mRNA to cDNA. Power SYBR Green PCR Master Mix (Takara Biotechnology, Dalian, China) was used to perform qPCR on the cDNA. Finally, the 2^–ΔΔCt^ method was used to evaluate the qPCR results. The GADPH gene was used as a control. The forward 5’-AGCTCCCTGAGGCAGAGTTG-3’ and reverse 5’-TCGTCAAGTGGGCTGTTAGG-3’primers were used for qPCR.

SERPINA3 expression levels in the urine and blood samples were measured using an ELISA kit (RayBio, USA) according to the manufacturer’s instructions. Blood serum and urine samples were obtained from healthy volunteers and patients and diluted 5,000 and 100 times, respectively.

### Measurement of chymase activity and the degranulation level of mast cells

Immunohistochemistry was performed according to conventional procedures. Anti-SERPINA3 antibody (ab205198, Abcam, 1:2000) was used as the primary antibody, and goat anti-rabbit IgG was used as the secondary antibody to investigate SERPINA3 expression differences between patients with DN and healthy individuals. An anti-mast cell chymase antibody (ab186417, Abcam, 1:250) was used as the primary antibody, and goat anti-rabbit IgG was used as the secondary antibody to reveal the difference in chymase activity between patients with DN and healthy controls. Fluorescence colocalization staining was used to study the localization of chymase and SERPINA3, which were detected using the anti-SERPINA3 antibody (ab205198, Abcam, 1:2000) and anti-mast cell chymase antibody (ab186417, Abcam, 1:250), respectively. Images were collected with a confocal microscope (LeicaSP5-FCS, Wetzlar, Germany), and colocalization correlation analysis was performed using ImageJ software with the plugin Coloc 2 (18).

To detect the density of mast cells and the level of degranulation in the renal tubular tissues of patients with DN, tubule tissue sections were stained with toluidine blue (Servicebio, G1032). Ten non-overlapping regions were randomly selected for each section and analyzed by two observers. The total number of positive and granulated mast cells stained with toluidine blue was calculated.

### Statistical analysis

All data are expressed as mean ± SEM. Statistical analyses were performed using GraphPad Prism, version 8 (GraphPad Software, San Diego, CA, USA). Significance was set at *P < 0.05, **P < 0.01, ***P < 0.001, and ****P < 0.0001.

## Results

### Identification and enrichment analysis of DEGs

**Figure 2:**
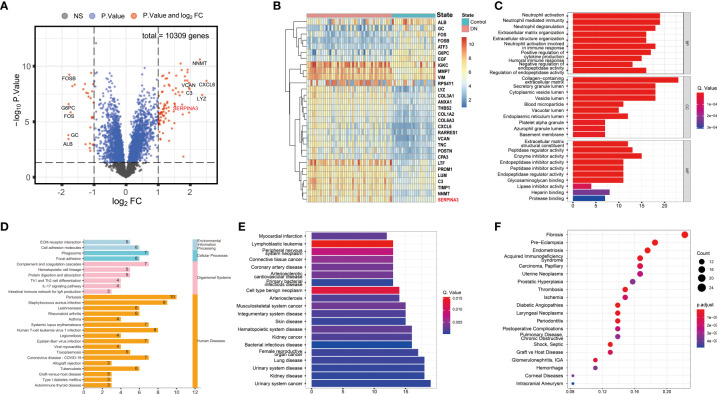
Differential expression and enrichment analyses results. **(A)** Volcano plot of DEGs. **(B)** Heatmap of the top 30 genes with the highest expression differences. **(C)** Barplot of GO enrichment analysis. Results were categorized into biological process (BP), cellular component (CC), and molecular function (MF). **(D)** Barplot of KEGG pathway enrichment analysis. The results were classified according to category information from the KEGG pathway database. **(E)** Barplot of DOSE enrichment analysis. **(F)** Dotplot of MeSH enrichment analysis. DEG, Differentially expressed gene; GO, Gene Ontology; KEGG, Kyoto Encyclopedia of Genes and Genomes; DOSE, Disease Ontology Semantic and Enrichment; MeSH, Medical Subject Headings.

Three datasets, GSE30529, GSE47184, and GSE104954, were batch normalized and merged. Unnormalized and normalized data were visualized (**Supplementary Figure S1**). A total of 113 DEGs were screened, including 86 upregulated and 27 downregulated DEGs, as shown in the volcano plot and heatmap (**Figures 2A, B**). GO, KEGG, DOSE, and MeSH enrichment analyses were performed to identify the biological processes and pathways related to DEGs. GO enrichment analysis showed that several biological processes (BP) related to neutrophil function were enriched, including neutrophil activation, neutrophil-mediated immunity, and neutrophil degranulation. The products of DEGs were associated with cellular components (CC), including cytoplasmic vesicles that can be transported to the extracellular matrix. Enzyme inhibitor activity was repeatedly enriched according to the molecular function (MF) terms (**Figure 2C**). KEGG pathway analysis showed that the DEGs were related to immune diseases and the immune system (**Figure 2D**). As for DOSE enrichment, the results showed that urinary system diseases were enriched, such as urinary system cancer, kidney disease, and kidney cancer (**Figure 2E**). Fibrosis was particularly enriched in terms of MeSH enrichment (**Figure 2F**).

### KEGG analysis, GSEA, and WGCNA showed enrichment in immune-related pathways

**Figure 3:**
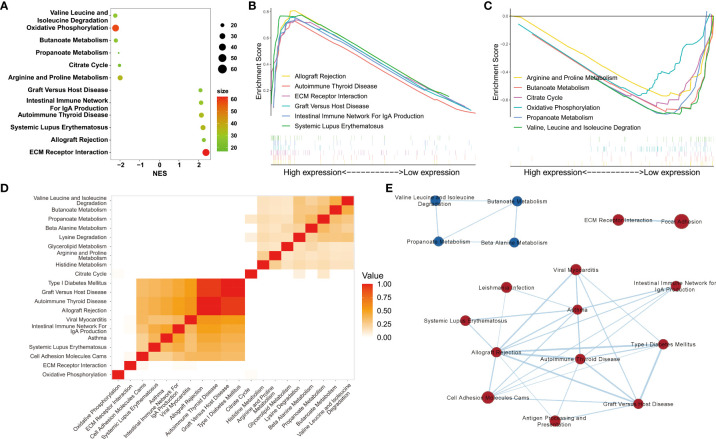
Gene set enrichment analyses. **(A)** The six pathways with the highest normalized enrichment scores (NES) in DN and control groups. **(B, C)** Visualization of the six pathways with the highest NES in the DN and control groups. **(D)** Heatmap and **(E)** enrichment of the results of leading edge analysis.

Among the 153 genes, 95 upregulated gene sets were significantly enriched in the DN group, 30 of which met the criteria of normalized P values < 0.05 and FDR < 0.25. Overall, 58 upregulated genes were significantly enriched in the control group, 22 of which met the criteria described above. Six pathways with the highest normalized enrichment scores in the DN and control groups were visualized (**Figures 3A-C**). Leading edge analysis showed that several pathways related to immune diseases were closely correlated with one another, such as type I diabetes mellitus, graft versus host disease, autoimmune thyroid disease, and allograft rejection. Heatmaps and enrichment maps were visualized (**Figures 3D, E**).

**Figure 4:**
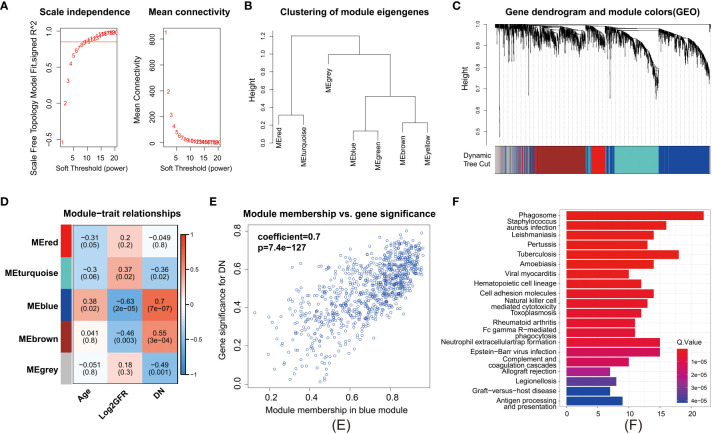
Weighted gene correlation network analysis. **(A)** Clustering of modules. **(B)** Clustering dendrogram of genes with dissimilarity based on topological overlap matrix. **(C)** Heatmap of the correlation between modules and clinical traits. Each row represents a module, and each column represents a trait. **(D)** Each cell contains the corresponding correlation and P value. Age, Log_2_GFR, and DN refer to age, logarithmic glomerular filtration rate, and traits of patients with DN. **(E)** Scatter plot of genes in the blue module. The horizonal axis represents module membership (MM). The vertical axis represents the importance of genes for DN. Pearson correlation coefficients of MM and gene significance and the P value are listed at the top of the plot. **(F)** KEGG pathway enrichment analysis of genes in the top 25% of MM.

To establish a gene regulatory network, power = 9 was selected for the soft threshold so that the scale-free network evaluation coefficient could reach 0.85 (**Figure 4A**). Then, a total of seven gene modules were obtained from the co-expression network (unpublished data) (**Figure 4B**). Five identical modules were retained when the dynamic pruning method was used (**Figure 4C**). Coefficients and the corresponding statistical significance between module eigengenes and clinical traits were calculated and visualized (**Figure 4D**). The blue module was most negatively related to the logarithmic GFR (Log_2_GFR) and positively related to the DN trait. Besides, it is known that GFR is the main marker of DN, which is negatively related to the progression of DN. In conclusion, the blue module was highly related to DN progression, with a correlation coefficient of 0.7 and P value of 7.4e-127 (**Figure 4E**). KEGG pathway enrichment analysis of module-important genes showed that the blue module was related to phagosomes, *Staphylococcus aureus* infection, and leishmaniasis (**Figure 4F**).

**Figure 5:**
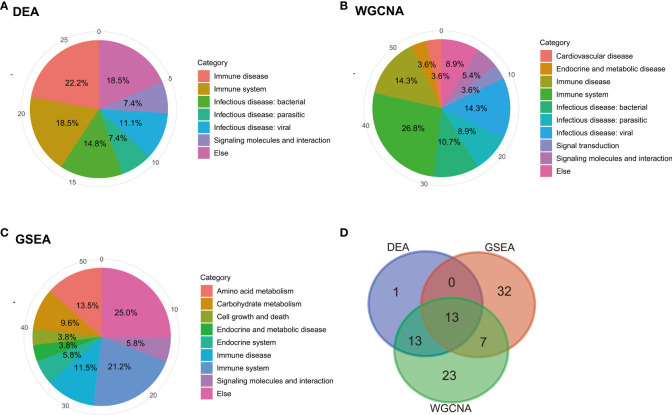
Categorization and intersection of pathway enrichment analysis. Pie charts of categorization of pathway enrichment analysis based on the results of **(A)** KEGG, **(B)** GSEA, and **(C)** WGCNA. **(D)** Venn diagram of pathways based on the three enrichment analysis methods: KEGG, GSEA, and WGCNA.

The pathways obtained from the three enrichment analysis methods were categorized based on their functional relevance (**Figure 5**). Terms relating to immune disease and the immune system accounted for a large proportion: 40.7% in DEGs (**Figure 5A**), 32.7% in GSEA (**Figure 5B**), and 43.2% in module-important genes (**Figure 5C**). Furthermore, a Venn diagram for pathways enriched according to KEGG analysis, GSEA, and WGCNA was constructed to identify 13 identical pathway items (**Figure 5D**), which are listed in **Table 2**. More than half of the cases were related to the immune system.

**Table 2:** Summary of 13 identical KEGG pathways from the intersection of the results of DEA, GSEA, and WGCNA.

Pathway	Major category	Minor category
Focal adhesion	Cellular process	Cellular community–eukaryotes
Cell adhesion molecules	Environmental information processing	Signaling molecule and interaction
ECM–receptor interaction		
Type I diabetes mellitus	Human diseases	Endocrine and metabolic disease
Viral myocarditis		Cardiovascular disease
Leishmaniasis		Infectious disease: parasitic
Asthma*		Immune disease*
Systemic lupus erythematosus*		
Graft-versus-host disease*		
Allograft rejection* Autoimmune thyroid disease*		
Intestinal immune network for IgA production*	Organismal systems	Immune system*
Complement and coagulation cascades*		

*These pathways are associated with immune processes.

### Immune-related hub gene selection and immune infiltration analyses

**Figure 6:**
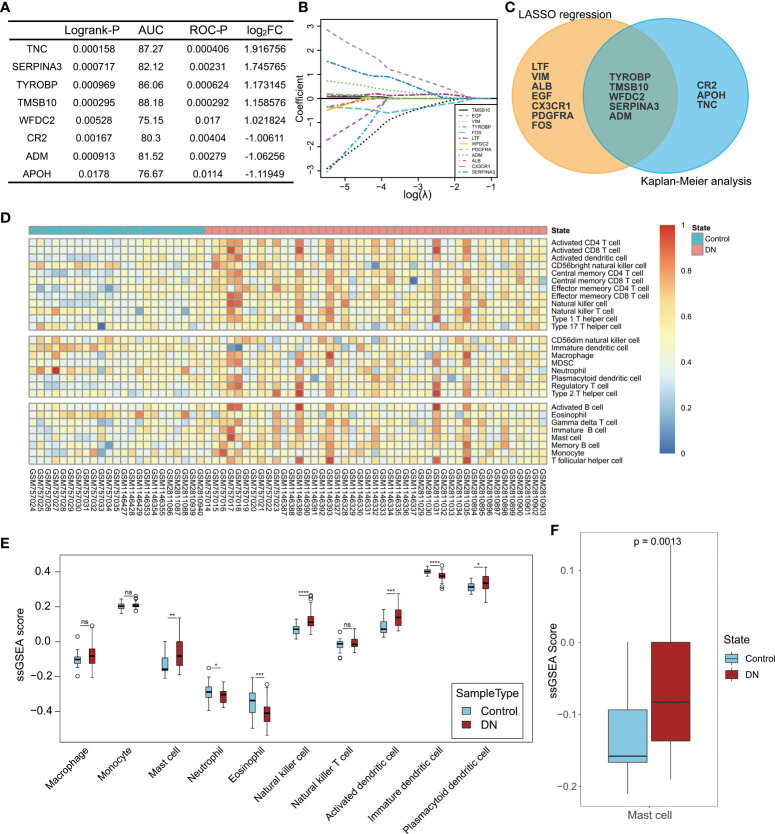
Hub gene selection and landscape of immune cell infiltration in DN. **(A)** Statistical indexes of Kaplan–Meier analysis for eight IRDEGs screened through dbPKD. Logrank-P value < 0.05 and ROC-P value < 0.05 were considered statistically significant. **(B)** Lasso regression analysis. The expression levels of 32 IRDEGs were used as input values for lasso selection. The final lasso regression model retained 12 IRDEGs. **(C)** Results of survival analysis and lasso regression screening were intersected to obtain five immune-related differentially expressed genes. **(D)** Heatmap of 22 subpopulations of immune cells between DN and normal kidney tubular tissue. **(E)** Difference in the estimated immune cell infiltration level between patients with DN and healthy controls. **(F)** Comparison of the estimated mast cell infiltration level between patients with DN and healthy controls. P value < 0.05 was considered statistically significant. *P < 0.05, **P < 0.01, ***P < 0.001 and ****P < 0.0001; ns, not statistically significant.

As mentioned above, changes in the immune system are strongly related to the occurrence and development of DN. To further elucidate the pathogenesis of DN at the molecular and cellular levels, 32 IRDEGs were identified by the intersection of 113 DEGs in DN and 2,483 IRGs downloaded from the ImmPort database. Then, 32 IRDEGs were submitted to dbPKD to perform Kaplan–Meier survival analysis from clinical data. Eight IRDEGs met the statistical requirements as potential prognostic markers (**Figure 6A**). Furthermore, 12 genes from the same 32 IRDEGs were selected as key variables in the process of constructing the lasso regression model (**Figure 6B**). Five identical IRDEGs were identified by taking the intersection. These genes not only acted as statistically significant IRDEGs but also functioned as lasso model variables (**Figure 6C**).

ssGSEA was performed to assess the extent of immune infiltration in the samples (**Figure 6D**). Here, the focus was on the role of innate immune cells in DN, and 10 cell types from 28 immune cells were selected: monocytes, macrophages, mast cells, neutrophils, eosinophils, natural killer cells, natural killer T cells, activated dendritic cells, immature dendritic cells, and plasmacytoid dendritic cells. The ssGSEA scores of the 10 cell types in the DN group were compared with those in the control group, as shown in **Figure 6E**. Except for monocytes, macrophages, and natural killer T cells, there were significant differences in the infiltration levels of the seven other types of cells in DN. Among the five identical IRDEGs obtained from the screening above, differential expression of SERPINA3 was the most significant, which was considered a potentially valuable molecule for further analysis. More importantly, when reviewing the function of IRDEGs on the online database *Uniprot*, it was found that the expression product of SERPINA3, alpha-1-antichymotrypsin, inhibits mast cell chymase, while other hub genes did not relate to immune cells with altered levels of infiltration functionally. Therefore, mast cells in the DN group were selected as potential associated immune cells. The statistical significance analysis of the difference in mast cells between the DN and control groups is shown in **Figure 6F**.

### High expression of SERPINA3 in the kidney tubules of patients with DN

**Figure 7:**
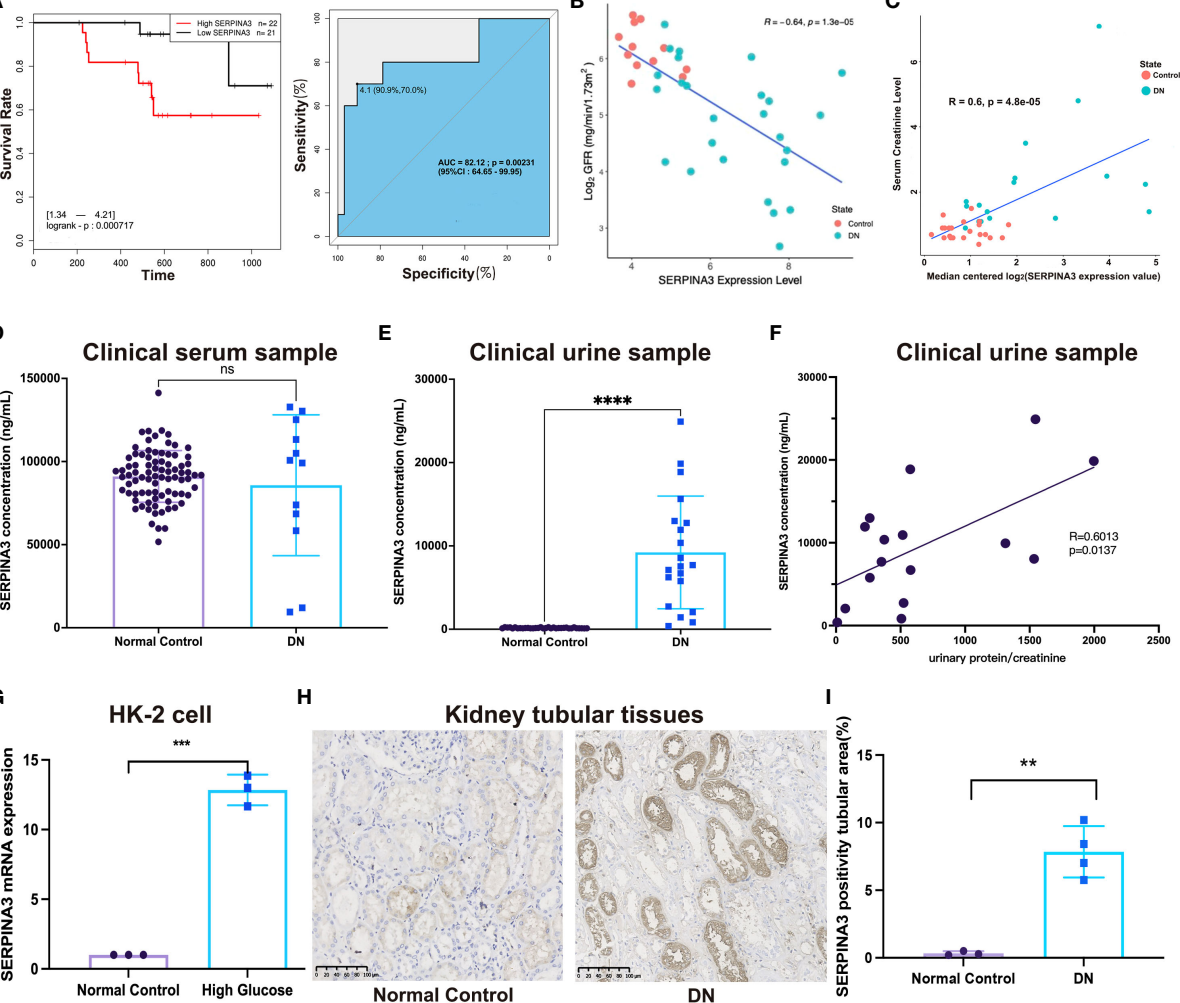
Validation of the relationship between SERPINA3 and DN. **(A)** Kaplan–Meier curve of SERPINA3 provided by dbPKD and the corresponding ROC curve. **(B)** Correlation between the SERPINA3 expression level and Log_2_GFR (GFR, glomerular filtration rate). **(C)** Correlation between median centered log_2_ (SERPINA3 expression value) and serum creatinine levels. Both GFR and serum creatinine level data were obtained from the database *Nephroseq*. **(D)** Comparison of the concentration of SERPINA3 proteins in serum samples between patients with DN and healthy controls. **(E)** Comparison of the concentration of SERPINA3 proteins in urine samples between patients with DN and healthy controls. **(F)** Correlation between the SERPINA3 protein concentration and urinary protein/creatinine levels. **(G)** The relative mRNA expression level of SERPINA3 in the HK-2 cells treated/untreated with high glucose levels was measured using qPCR. **(H)** SERPINA3 immunohistochemical staining in kidney tubular sections from patients with DN and healthy controls. Scale bars = 25 µm. **(I)** Semi-quantitative analyses of the immunohistochemically stained SERPINA3-positive tubule area. **P < 0.01, ***P < 0.001, and ****P < 0.0001; ns, no significance.

Next, bioinformatic analyses results were validated with experiments. Kaplan–Meier analysis showed that high SERPINA3 expression was also significantly correlated with poor overall survival in patients with DN (**Figure 7A**). The GFR and serum creatinine levels are markers of DN progression. The former was negatively correlated with DN, whereas the latter was positively correlated with DN in clinical diagnosis. Pearson correlation analysis showed that SERPINA3 expression was negatively correlated with Log_2_GFR (**Figure 7B**) and positively correlated with serum creatinine levels (**Figure 7C**), which suggested that SERPINA3 was highly related to DN.

To examine SERPINA3 protein levels in the blood and urine, clinical samples from patients with DN were collected and tested. The expression levels of SERPINA3 in the blood of the patients with DN were not significantly different from those in the blood of healthy controls (**Figure 7D**). However, SERPINA3 expression levels in urine samples from patients with DN were significantly higher than those in urine samples from healthy controls (**Figure 7E**), and the expression level of SERPINA3 in urine was positively correlated with urinary protein/creatinine levels (**Figure 7F**).

The expression of SERPINA3 in human kidney tubular cell lines and kidney tubular tissues was examined using RT-PCR and immunohistochemical staining. RT-PCR results showed that the relative SERPINA3 mRNA expression levels in proximal tubular epithelial cells (HK-2 cells) stimulated with high glucose to mimic diabetes *in vitro* were significantly higher than those in the normal control group (**Figure 7G**). SERPINA3 expression was further investigated in pathological tissues using immunohistochemical staining. The results indicated that the expression of SERPINA3 in kidney tubular tissues was substantially higher in patients with DN than in healthy controls (**Figure 7H**), and the difference was statistically significant based on semi-quantitative analysis of the SERPINA3 positive tubule area (**Figure 7I**). These results verified that SERPINA3 was significantly differentially expressed in the renal tubules of patients at the cellular, tissue, and clinical levels. The correlation between SERPINA3 and DN progression showed that SERPINA3 has the potential to serve as a biomarker of DN. DN progression can be determined by the expression level of SERPINA3 in urine.

### Validation of the correlation of mast cells and chymase with DN

**Figure 8:**
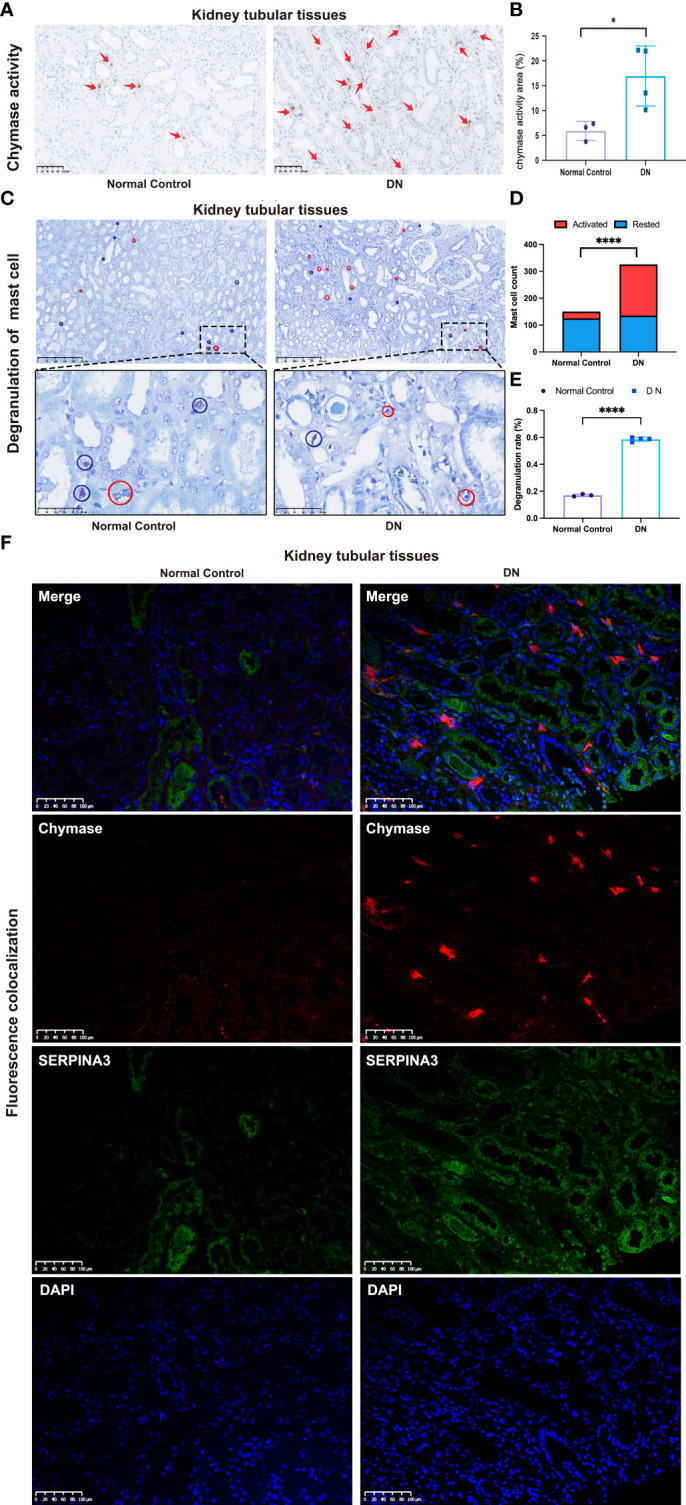
Correlation between SERPINA3 expression levels and chymase activity in mast cells. **(A)** Images of immunohistochemical staining for chymase activity in kidney tubular tissue samples from patients with DN and healthy controls. Scale bars = 100 µm. **(B)** Semi-quantitative analyses of the chymase activity area. **(C)** Observation of mast cell degranulation in the renal tubular tissue of patients with DN and healthy controls by toluidine blue staining. Red circles indicate cells in a degranulated state, while blue circles indicate cells in a resting state. Scale bars = 250 µm (images above) and scale bars = 50 µm (images below). Microscopes were used at magnifications of 100× and 500×, respectively. **(D)** Histogram of the number of activated and resting mast cells in the DN and control groups. **(E)** Proportion of mast cell degranulation observed in patients with DN and healthy controls determined by toluidine blue staining. Data shown are the mean ± SEM (n = 4). **(F)** Colocalization of SERPINA3 (green) and chymase (red) in tubule tissue. *P < 0.05 and ****P < 0.0001.

Immunohistochemistry analyses were used to investigate the distribution and differences in chymase levels in the DN and normal groups. The level and area of activated chymase were significantly higher in the DN group than in the control group (**Figures 8A, B**). Toluidine blue staining was performed to analyze the distribution, number, and level of mast cell degranulation in the kidney tubular tissues. In patients with DN, mast cells were diffusely distributed in the renal interstitium, especially around the renal tubules and glomeruli (**Figure 8C**). The number and density of mast cells were increased in patients with DN compared to healthy controls, with a density of 5.0 (1.9~7.2) mast cells/mm^2^ in healthy controls and 8.2 (5.1~11.8) mast cells/mm^2^ in patients with DN (**Figure 8D**). Mast cell degranulation was clearly observed in toluidine blue-stained sections, with 16.99% of mast cells in healthy controls showing degranulation, whereas the level of mast cell degranulation was significantly increased in patients with DN, reaching 58.58% (**Figure 8E**). Immunofluorescence analysis confirmed that chymase-positive cells were mostly concentrated in the renal peritubular region and showed high fluorescence intensity in DN samples. In contrast, SERPINA3 was localized in the extracellular zone in the renal tubular region as a secreted protein and diffused around the tubular tissue. The obtained images suggested that the SERPINA3-positive region covered the chymase-positive region. These results indicated that SERPINA3 may play a role in blocking mast cell chymase activity (**Figure 8F**).

## Discussion

DN is a serious disease in clinical practice, but its pathogenesis has not yet been fully elucidated. Previously, we discovered that PPBP is a novel DN biomarker of podocyte injury (19). Moreover, we found that tubular injury plays an important role in DN. Therefore, in the present study, we aimed to identify early markers of tubular injury in DN by performing comprehensive bioinformatics analysis. First, three methods, differential expression analysis, GSEA, and WGCNA, were used to describe the characteristics of DN. The results were surprisingly consistent, indicating that DN is highly related to the immune system, which prompted us to screen for IRDEGs and perform immune infiltration analysis. SERPINA3 was identified as a potential target gene by screening IRDEGs and combining clinical data and machine learning methods. To date, the physiological function of SERPINA3 is unclear, and only a few reports have shown that it can inhibit the chymase activity of mast cells, which convert angiotensin-1 to active angiotensin-2. In contrast, immune infiltration analysis showed that the proportions of different states of mast cells were significantly related to the state of patients with DN. Therefore, we inferred a correlation between SERPINA3 and chymase secreted from mast cells, which jointly regulate the progression of diabetic renal tubular injury.

As a member of the serine protease inhibitor (SERPIN) family, SERPINA3 plays an important role in regulating cellular processes, such as angiogenesis, apoptosis, fibrosis, oxidative stress, and inflammatory response (20). Our RT-PCR results showed that SERPINA3 is highly expressed in HK-2 cells treated with high levels of glucose *in vitro*. However, the physiological and pathological roles of SERPINA3 in the kidneys are not yet understood. SERPINA1 and SERPINA3 have been reported to co-localize with lysozyme in proximal renal tubular cells, especially in patients with immunonephropathy (21). In addition, SERPINA3 has been found in renal tumor cells, such as nephroblastoma, renal carcinoma, and congenital mesodermal nephroma cells (22). During the transition from acute kidney injury to chronic kidney disease, proteinuria and renal fibrosis progressively increased in rats. SERPINA3 was shown to be transferred from the cytoplasm to the tubular apical membrane, causing abnormal levels of SERPINA3 in urine, suggesting that SERPINA3 may serve as a potential biomarker of early detection of AKI to CKD transition (23). Within the serine protease inhibitor family, SERPINB2, SERPINA1, and SERPINA4 have also received widespread attention. The *SERPINB2* gene encoding the protein plasminogen activator inhibitor 2 is associated with the delayed development of DN and chronic kidney disease. It can enhance the expression of the chemokine CCL2 in renal tubular cells and plays a role in kidney aging, injury, and repair by activating macrophage phagocytosis and inhibiting macrophage migration (24, 25). The *SERPINA1* gene, which encodes the protein α1-antitrypsin, can improve renal function, reduce renal tubular necrosis and macrophage infiltration in the abdominal cavity after bilateral renal ischemia/reperfusion in mice, and plays a protective role in the kidneys (26). SERPINA4 in the db/db DN mouse model can mediate the inflammatory response and oxidative stress, and reduce angiogenesis in the kidneys and the degree of tubule injury and fibrosis (27).

Chymase secreted by mast cells is involved in vasoactive peptide generation, extracellular matrix degradation, and glandular secretion regulation (28, 29). Chymase is associated with diabetic renal tubular injury. Chymase was found to activate TGF-β in cultured fibroblasts, thereby inducing renal fibrosis and leading to DN. In db/db mice, chymase upregulated renal angiotensin II (AngII) generation, and this is known as the chymase-dependent AngII-generating system. The upregulated chymase-dependent AngII-generating system in human diabetic kidneys increases the urinary albumin excretion rate, which is an important indicator of DN development, suggesting that chymase may be related to DN development (30). Further studies have shown that the chymase-dependent AngII-generating system upregulates NOX4-dependent oxidative stress to induce podocyte damage. Podocyte damage is relieved in db/db mice when podocytes are treated with a chymase inhibitor. These results suggest that podocyte injury in the glomerulus is caused by a chymase-dependent AngII-generating system (28). Regarding the regulation of chymase activity, SERPINA3 can inhibit several serine proteases secreted from neutrophils and mast cells, including chymase (31). Through enzymatic reactions, chymase can activate stem cell factors. Activated stem cell factors can stimulate mast cell development and proliferation, resulting in an increased number of mast cells (28).

Mast cells are granulocytes rich in secretory granules that contain a variety of inflammatory mediators, including inflammatory cytokines, endothelin, growth factors, and proteolytic enzymes (32). Mast cells in the human body can be classified into two subtypes, MC_T_ and MC_TC_, depending on whether they produce tryptase only, or both tryptase and chymase (33). Monoclonal antibodies against tryptase can be used to identify mast cells in tissues. Antibodies against chymase are often used to recognize mast cells by immunohistochemistry (34). In the pathological examination of DN, mast cells are widely distributed in the interstitial tissue, especially in the area around the renal tubules, blood vessels, and glomerulus, and partially infiltrate the atrophic tubular walls. With the progression of DN, the density and degranulation level of mast cells increase significantly, indicating that these increases are significantly correlated with renal tubular interstitial injury (33). In a rat model of type I DN, mast cells were observed in the kidneys of DN rats, and both the number and degranulation levels of the mast cells were significantly higher than those in the normal group. When the anti-fibrosis compound Trinistra was used to inhibit mesenteric vascular fibrosis, the number of mast cells of the MC_TC_ subtype decreased, while the number of mast cells of the MC_T_ subtype did not change (32). This suggests that mast cells, especially the MC_TC_ subtype, are closely associated with DN development.

**Figure 9:**
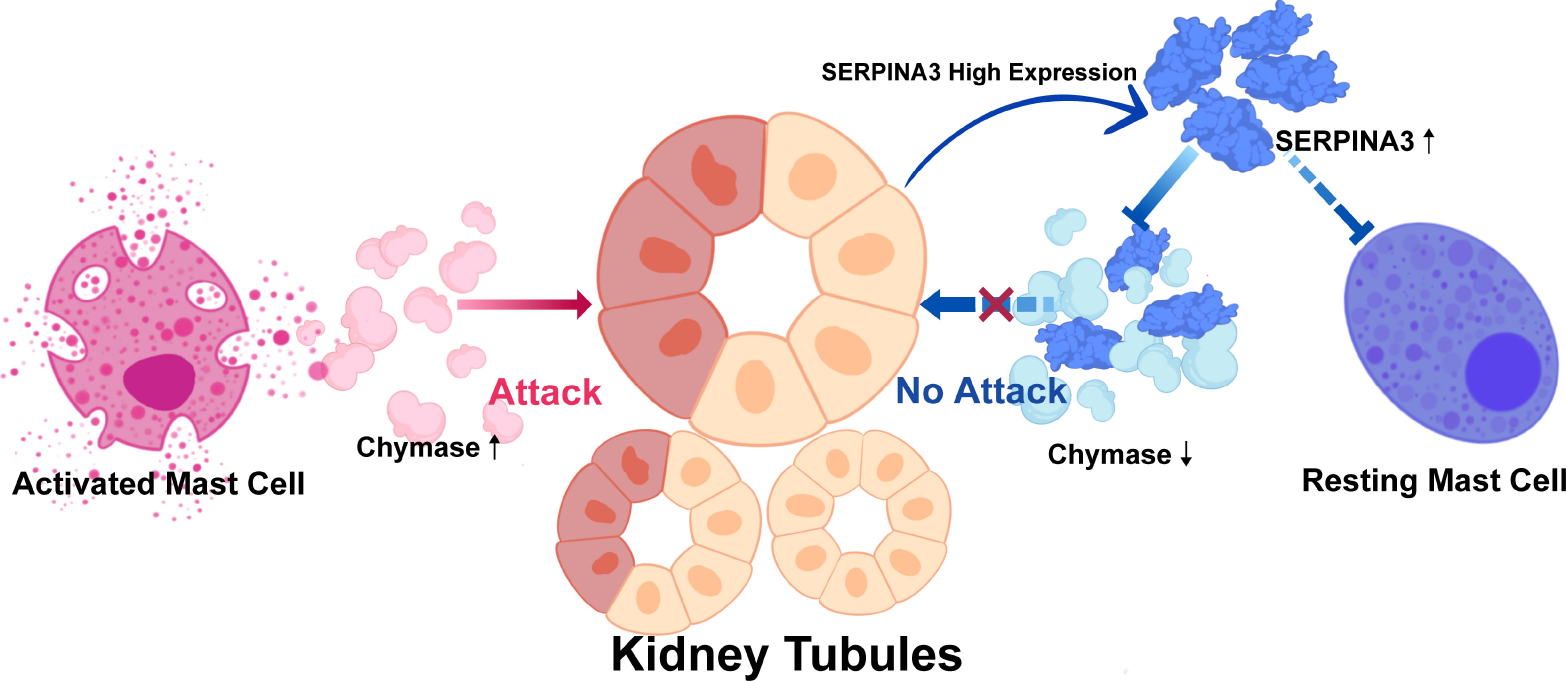
Protective mechanism of SERPINA3 in kidney tubules in response to damage by chymase.

In summary, we hypothesized that SERPINA3, a protective molecule that prevents renal tubular injury in patients with DN, could inhibit mast cell proliferation and activation by downregulating chymase activity (**Figure 9**). To examine our hypothesis, several experiments involving SERPINA3, chymase, and mast cells were performed. High expression of chymase was found in the DN group *via* immunohistochemical analyses. In addition, the number and degranulation level of mast cells were significantly increased in the DN group, which validated the results of the immune infiltration analyses. As for the expression of SERPINA3, RT-PCR and immunohistochemistry analyses revealed that a high level of SERPINA3 was accompanied by diabetic renal tubular injury *in vitro*. The increasing number and degranulation level of mast cells partially account for the kidney injury. Overexpressed chymase, which marks activated mast cells, can attack kidney cells and deteriorate diabetic renal tubular injury. In summary, we inferred that in order to protect themselves from chymase, kidney cells upregulate the expression of SERPINA3, which weakens the harmful effect of chymase on kidney cells by inhibiting chymase activity (**Figure 9**).

## Data availability statement

The datasets presented in this study can be found in online repositories. The names of the repository/repositories and accession number(s) can be found in the article/**Supplementary Material**.

## Ethics statement

The studies involving human participants were reviewed and approved by ethics review committee of Guangdong Provincial People’s Hospital. The patients/participants provided their written informed consent to participate in this study. Written informed consent was obtained from the individual(s) for the publication of any potentially identifiable images or data included in this article.

## Author contributions

QL, ZF, and YG were responsible for the overall design and investigation. ZF, YG, and NJ performed data analysis. ZF, YG, NJ, FZ, SL, and QL were responsible for manuscript writing and participated in discussion of the results. All authors contributed to the article and approved the submitted version.

## Funding

This work was supported by grants from the National Natural Science Foundation of China [No. 81770494 (QL), No. 32271360 (QL) and No. 81870508 (SL)]; the Natural Science Foundation of Guangdong Province, China [No. 2021A1515010040 (QL) and No. 2022A1515012374 (SL)], the Guangdong Province High-level Hospital Construction Project [No. DFJH201901 (SL)], and the Innovation and Technology Commission, Hong Kong [No. 2019B121205005 (SL)].

## Conflict of interest

The authors declare that the research was conducted in the absence of any commercial or financial relationships that could be construed as a potential conflict of interest.

## Publisher’s note

All claims expressed in this article are solely those of the authors and do not necessarily represent those of their affiliated organizations, or those of the publisher, the editors and the reviewers. Any product that may be evaluated in this article, or claim that may be made by its manufacturer, is not guaranteed or endorsed by the publisher.
