# Supplementary material for: Immune-related SERPINA3 as a biomarker involved in diabetic nephropathy renal tubular injury
Source: Front Immunol. 2022 Oct 11;13:979995. doi: 10.3389/fimmu.2022.979995 (PMC9592916; doi:10.3389/fimmu.2022.979995)
Supplement: Supplementary file 1 [file DataSheet_1.docx]

Supplementary Material

# Supplementary Figures


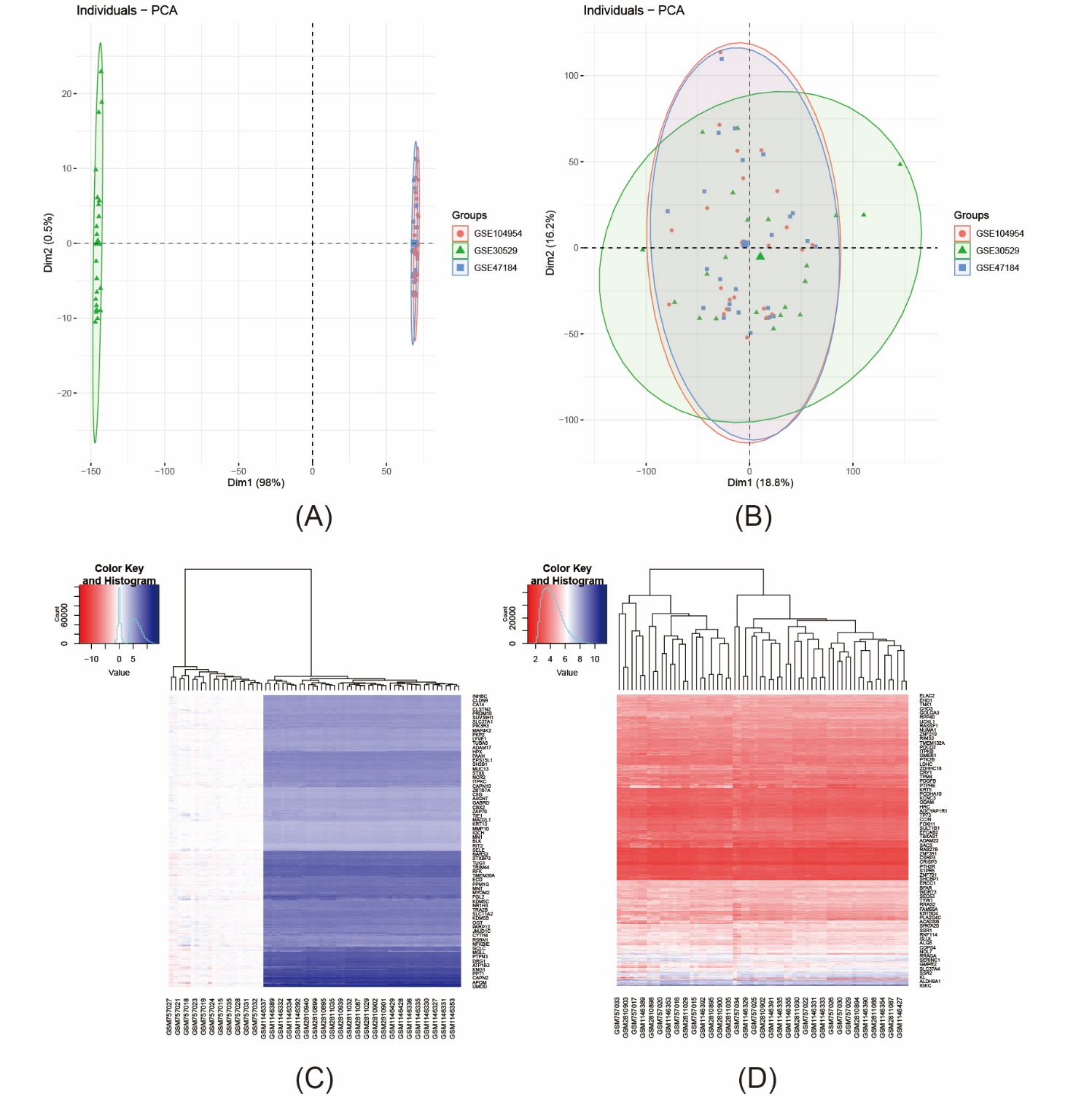


**Figure S1: Quality control of data**. After datasets GSE30529, GSE96804 and GSE104954 were merged, batch effect of merged data was eliminated. PCA scatter plots (A) (B) and heatmaps (C) (D) were used to characterize the effect of before (A,C) and after(B,D) elimination of batch effect.


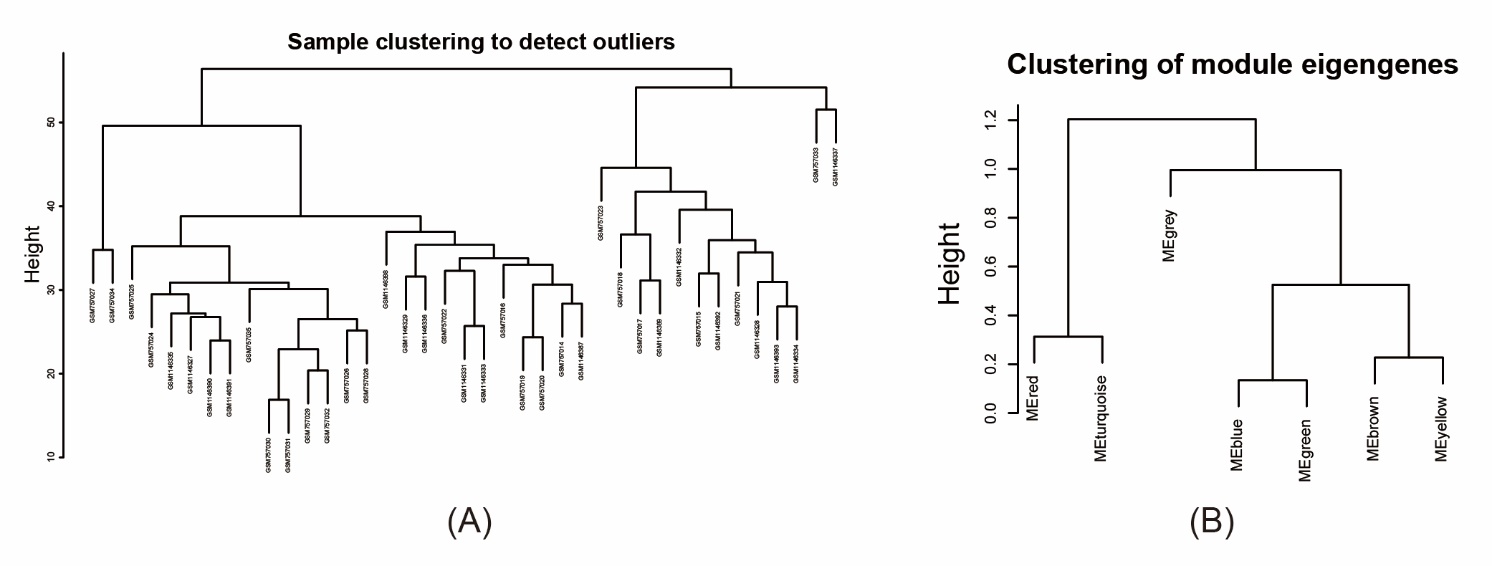


**Figure S2: Quality control of WGCNA.** (A) Outlier samples detection of WGCNA. No outliers were removed. (B) Clustering tree of module detected by dynamic tree cut. Seven modules were detected firstly. After clustered, blue and green module were merged together into the blue one, while brown and yellow module were merged together into the brown one.
